# Treatment-Emergent Neuroendocrine Prostate Cancer: A Clinicopathological and Immunohistochemical Analysis of 94 Cases

**DOI:** 10.3389/fonc.2020.571308

**Published:** 2021-02-01

**Authors:** Qingfu Zhang, Yunan Han, Yao Zhang, Dan Liu, Jian Ming, Bo Huang, Xueshan Qiu

**Affiliations:** ^1^Department of Pathology, The First Affiliated Hospital and College of Basic Medical Sciences, China Medical University, Shenyang, China; ^2^Division of Public Health Sciences, Department of Surgery, Washington University School of Medicine, St. Louis, MO, United States; ^3^Department of Breast Surgery, The First Affiliated Hospital of China Medical University, Shenyang, China; ^4^Department of Pathology, General Hospital of Northern Theater Command, Shenyang, China; ^5^Department of Pathology, The Liaoning Cancer Hospital & Institute of China Medical University, Shenyang, China

**Keywords:** treatment-emergent neuroendocrine prostate cancer, castration-resistant prostate cancer, small cell carcinoma, immunohistochemistry, SEER program

## Abstract

**Purpose:**

This study aimed to evaluate the pathological characteristics, immunophenotype, and prognosis of treatment-emergent neuroendocrine prostate cancer (T-NEPC).

**Materials and Methods:**

We collected 231 repeated biopsy specimens of castration-resistant prostate cancer (CRPC) cases between 2008 and 2019. We used histopathological and immunohistochemical evaluations of Synaptophysin (SYN), ChromograninA (CgA), CD56, androgen receptor (AR), and prostate**-**specific antigen (PSA) to screen out T-NEPC cases. Multivariate analyses were performed to identify factors in the prognosis of T-NEPC. Further, the results were verified in the Surveillance, Epidemiology, and End Results (SEER) program.

**Results:**

Among the 231 CRPC cases, 94 (40.7%) cases were T-NEPC. T-NEPC were more likely to present with negative immunohistochemistry for AR (30.9%) and PSA (47.9%) than that of CRPC (8.8% and 17.5%, respectively). Kaplan-Meier analysis revealed that patients with T-NEPC (median overall survival [OS]: 17.6 months, 95% CI: 15.3–19.9 months) had significantly worse survival compared with usual CRPC patients (median OS: 23.6 months, 95% CI: 21.3-25.9 months, log-rank *P* = 0.001), especially in metastasis cases (median OS: 15.7 months, 95% CI: 13.3-18.0 months) and patients with small cell carcinoma component (median OS: 9.7 months, 95% CI: 8.2–11.2 months). Prostate adenocarcinoma with diffuse NE differentiation (median OS: 18.8 months, 95% CI: 15.3–22.3 months) had poor outcome than those with usual CRPC (*P* = 0.027), while there was no significant change in the focal NE differentiation (median OS: 22.9 months, 95% CI: 18.1–27.7 months, *P* = 0.136). In the unadjusted model, an excess risk of overall death was observed in T-NEPC with PSA negative (HR = 2.86, 95% CI = 1.39–6.73). Among 476 NEPC cases in the SEER database from 2004 to 2017, we observed a higher hazard of overall death in patients aged 65 years and older (HR = 1.35, 95% CI = 1.08–1.69), patients with PSA ≤ 2.5 ng/ml (HR = 1.90, 95%CI = 1.44–2.52), patients with PSA 2.6–4.0 ng/ml (HR = 2.03, 95%CI = 1.38–2.99), stage IV tumor (HR = 2.13, 95%CI = 1.47–3.08) and other races (HR = 1.85, 95%CI = 1.17–2.94) in total NEPC, adjusting for confounders. Similar hazard ratios were observed in pure NEPC, while there was no significant results among prostate adenocarcinoma with NE differentiation tumors.

**Conclusion:**

T-NEPC was associated with an unfavorable prognosis, negative immunohistochemistry for PSA in T-NEPC and serum PSA level ≤ 4 ng/ml had a worse prognosis. Urologists and pathologists should recognize the importance of the second biopsy in CRPC to avoid unnecessary diagnosis and treatment delays.

## Introduction

Treatment-emergent neuroendocrine prostate cancer (T-NEPC) mainly occurs in the advanced castration-resistant prostate cancer (CRPC), which is caused by the transformation of ordinary prostate adenocarcinoma after androgen-deprivation therapy (ADT) (1). The main clinical manifestations of T-NEPC include low PSA level, high tumor metastasis load, and rapid resistance to ADT (2). T-NEPC is an aggressive variant of CRPC, with a poor prognosis. Most T-NEPC patients die within 1 to 2 years after diagnosis, accounting for approximately 25% of CRPC deaths (3). The clinical acquaintance of conversion to T-NEPC after resistance to endocrine therapy in prostate adenocarcinoma is insufficient, and secondary biopsies are not routinely performed, cause to the missed diagnosis of T-NEPC. At present, there is still no pathological consensus definition of T-NEPC. The morphological characteristics of T-NEPC are similar to poorly differentiated prostate adenocarcinoma, so the incidence of T-NEPC is greatly underestimated (4). In recent years, with the widespread use of novel, highly potent androgen receptor-targeted therapies (eg, Arbiton, MDV3100), the incidence of T-NEPC may also rise significantly. T-NEPC is not sensitive to ADT treatment, and the treatment is different from adenocarcinoma. Once T-NEPC is diagnosed, systemic chemotherapy based on etoposide combined with cisplatin (EP) or carboplatin(CE) should be performed as soon as possible, as well as radiotherapy, and other potentially effective treatment includes targeted drugs such as Aurora kinase A (AURKA) kinase inhibitors, anti-EGFR and mTOR inhibitors (5, 6) if necessary. Therefore, early diagnosis and treatment are of great significance to improve the patient’s survival benefit. But there are very few clinical series studies to date, and the clinical characteristics and prognosis of T-NEPC are not very clear. Therefore, we analyzed the clinicopathologic characteristics and outcomes of T-NEPC, intending to improve the understanding of T-NEPC among clinicians and pathologists.

## Materials and Methods

### Case Selection

A total of 231 cases of CRPC were collected from the First Affiliated Hospital of China Medical University, General Hospital of Northern Theater Command, and the Liaoning Cancer Hospital & Institute of China Medical University between 2008 and 2019. Overall survival (OS) was defined as the time interval between the date of CRPC or T-NEPC diagnosis and the date of death or last follow-up. By the Declaration of Helsinki, written informed consent was obtained from all participants. This study was approved by the Ethics Committee of China Medical University.

### Immunohistochemistry

All the specimens had been fixed by 10% formalin and paraffin-embedded routinely. Four µm thickness tissue sections from blocks were stained with hematoxylin and eosin (H&E) and immunohistochemical staining (Syn, CgA, CD56, AR, and PSA). Immunohistochemistry was performed by the avidin-biotin-peroxidase complex method (MaiXin Inc, China, prediluted) according to the manufacturer’s instruction.

### Immunohistochemical Evaluation

Two independent, blinded investigators, XSQ and QFZ evaluated all the slides. AR immunopositivity was located in the nucleus, CD56 immunopositivity was located in both the cell membrane and cytoplasm, PSA, SYN, and CgA immunopositivity were located in the cytoplasm. At least 10% of tumor cells showed at least one NE marker positive was considered as NE differentiation. According to the staining intensity, the expression level of immunohistochemical staining was scored as: 0 point (negative), 1 point (weak expression), 2 points (moderate expression-strong expression); staining cell percentage evaluation score: 0 point (0%–10%), 1 point (10%–50%), 2 points (51%–100%). The scores of staining intensity and the percentage of staining cells were multiplied, and the final score was >1 point was positive.

### SEER Cohort

**Figure 1:**
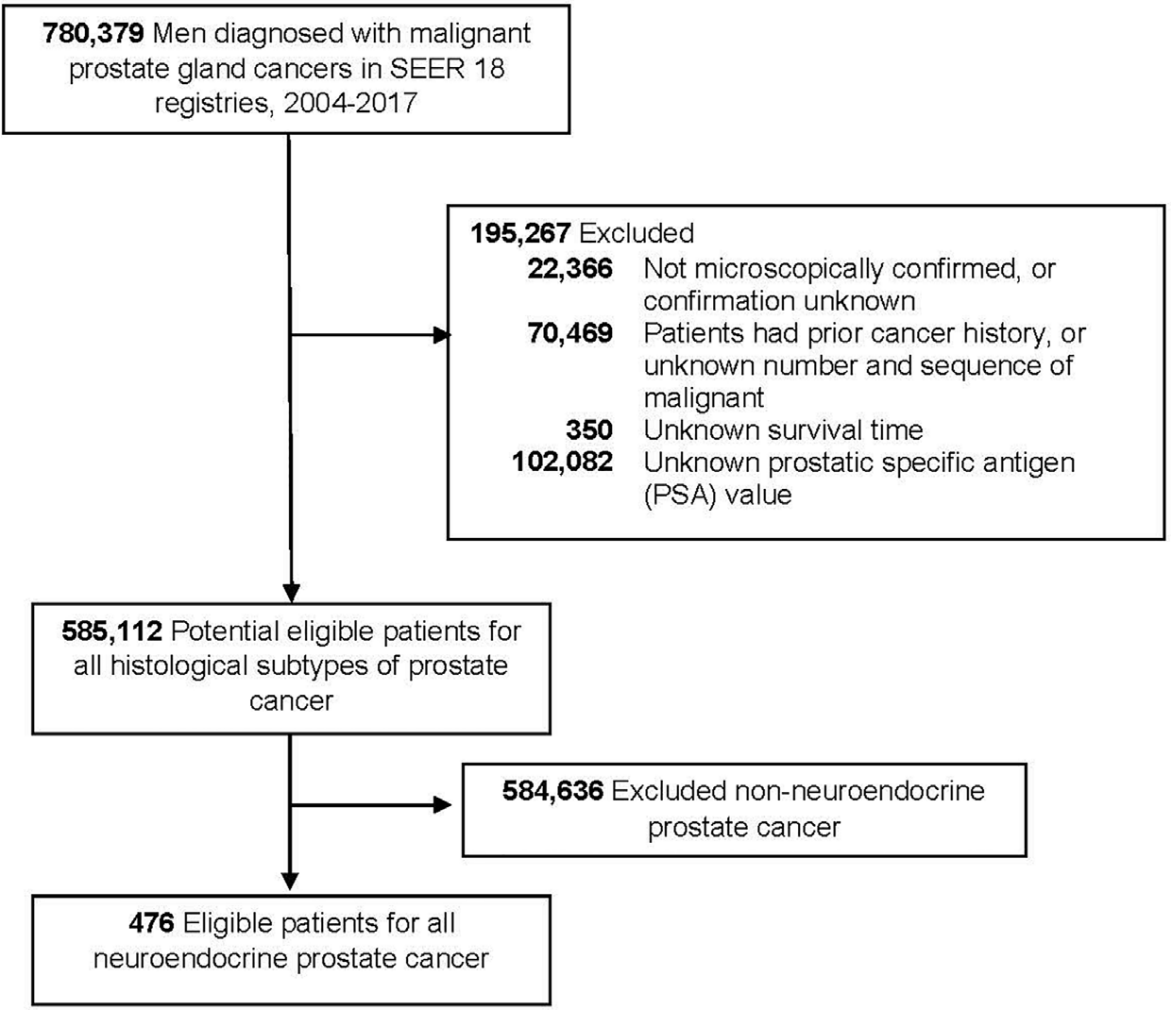
Flowchart of eligible individuals, surveillance, epidemiology, and end results (SEER) 18 registries, 2004–2017.

We used the SEER database (released in April of 2020) to identify eligible NEPC cases. This study included men diagnosed with malignant prostate adenocarcinomas between 2004 and 2017 (n = 780,379) because the SEER database did not collect PSA information until 2004. We excluded non-microscopically confirmed cases, patients with a prior cancer history, unknown survival time, men without PSA level. The final analytic sample included 476 NEPC cases **(** **Figure 1****)**. Due to the use of de-identified data, this study was determined as exempt by the Institutional Review Board of the First Hospital of China Medical University.

### Statistical Analysis

Chi-squared tests were applied to analyze the clinicopathological characteristics of subgroups. Survival curves were plotted according to the Kaplan-Meier method and compared using the log-rank test in a univariate analysis. Multivariate analysis was performed using the Cox proportional hazards model. The estimated risks for overall survival (OS) were calculated as hazard ratios (HRs) with 95% confidence intervals (CIs). All statistical analyses were performed using SAS (version 9.4; SAS Institute, Cary, NC) or SPSS 22.0. Statistical significance was assessed as two-sided *P<*0.05.

## Results

### Clinicopathological Features

Of the 231 cases, ages ranged from 51 to 82 years (mean, 68.5 years). The tumor size ranged from 1.5 to 8 cm (mean, 2.6 cm). 40.7% (n = 94) cases were T-NEPC. The median time from the initial diagnosis of prostate adenocarcinoma to the diagnosis of T-NEPC was 24.3 months (19.4–62.8 months). After T-NEPC diagnosis, 53 (56.4%) cases were treated with radiotherapy or chemotherapy. 57 (60.6%) cases appeared metastasis, among which 31 cases (54.4%) were bone metastasis, 13 (22.8%) cases were lymph nodes metastasis and 22 (38.6%) cases present organ (Brain, Lung, Liver. etc) metastasis, 12 (21.1%) cases were multiple metastases. 190 (82.3%) cases were AR positive, all were moderate-strong positive, 162 (70.1%) cases were PSA positive, of which 26 cases were weak positive. Among T-NEPC cases, 54 (57.4%) cases were CgA positive, 79 (84.0%) cases were SYN positive, 39 (41.5%) cases were CD56 positive. 31 (33.0%) cases were positive for only 1 neuroendocrine marker, 41(43.6%) were positive 2 neuroendocrine markers, and 22 cases (23.4%) were positive for both 3 neuroendocrine markers.

### Histological Findings

**Figure 2:**
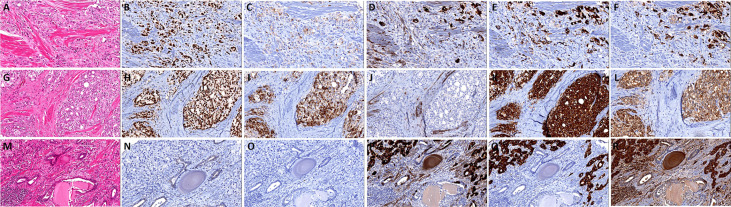
The histomorphology and immunohistochemistry of prostate adenocarcinoma with focal/diffuse increased neuroendocrine differentiation. **(A)** Prostate adenocarcinoma with focal increased neuroendocrine differentiation, the tumor cells show **a**ndrogen receptor (AR) **(B)** and prostate**-**specific antigen (PSA) **(C)** positive, and focal express CD56 **(D)**, Syn **(E)**, and CgA **(F)**; **(G)** prostate adenocarcinoma with diffuse neuroendocrine differentiation, the tumor cells show AR **(H)** and PSA **(I)** positive, and negative express CD56 **(J)**, but Syn **(K)**, and CgA **(L)** diffuse positive; **(M)** prostate adenocarcinoma with diffuse neuroendocrine differentiation, the tumor cells show AR **(N)** and PSA **(O)** negative, and diffuse express CD56 **(P)**, Syn **(Q)** and CgA **(R)**.

**Figure 3:**
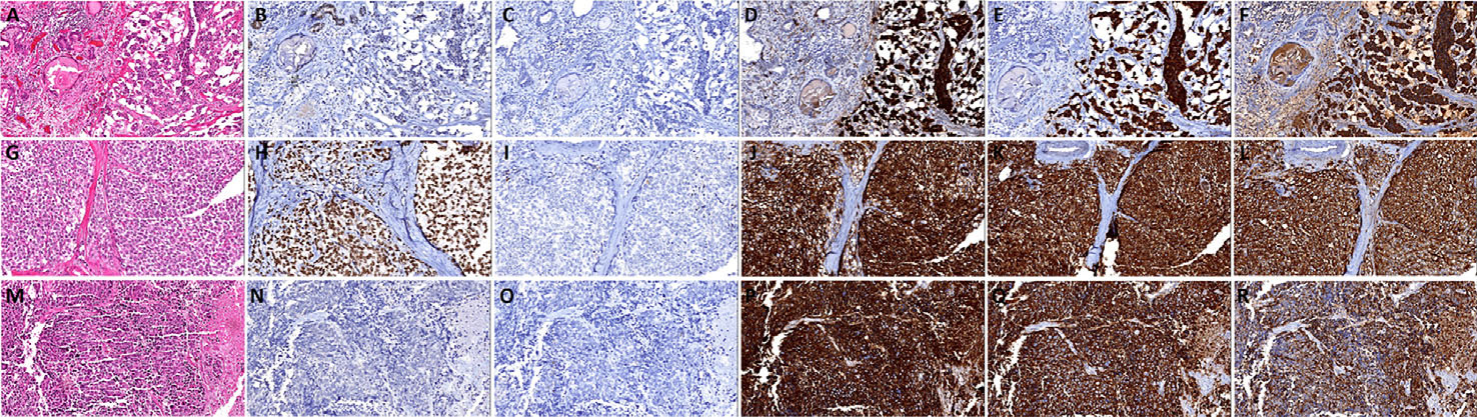
The histomorphology and immunohistochemistry of prostate highly differentiated neuroendocrine cancer and poorly differentiated neuroendocrine carcinoma. **(A)** Highly differentiated neuroendocrine cancer, the tumor cells show **a**ndrogen receptor (AR) **(B)** and prostate**-**specific antigen (PSA) **(C)** negative, and diffuse express CD56 **(D)**, Syn **(E)**, and CgA **(F)**; **(G)** Prostate large-cell neuroendocrine carcinoma, the tumor cells show AR **(H)** positive, and negative express PSA **(I)**, diffuse express CD56 **(J)**, Syn **(K)**, and CgA **(L)**; **(M)** Prostate small-cell carcinoma, the tumor cells show AR **(N)** and PSA **(O)** negative, and diffuse express CD56 **(P)**, Syn **(Q)** and CgA **(R)**.

The cases were divided into five groups according to morphological and the expression extent of neuroendocrine markers: 1. Usual CRPC, there were 137 cases, of which 125 cases were AR positive and 113 cases were PSA positive. 2. Prostate adenocarcinoma with focal increased neuroendocrine differentiation **(** **Figures 2A–F****)**, confirmed by immunohistochemistry with neuroendocrine differentiation, but did not meet the criteria of group 3, of which 23 cases were matched, all 23 cases were AR positive, and 17 cases were PSA positive. And there were 16 cases with Gleason score >7, 7 cases with Gleason score ≤7. 3. Prostate adenocarcinoma with diffuse neuroendocrine differentiation (typical prostate acinar adenocarcinoma or prostate ductal adenocarcinoma with at least 1 neuroendocrine marker >50% positive) **(** **Figures 2G–R****)**, and of which 34 cases were matched, 27 cases were AR positive, and 23 cases were PSA positive. In addition, there were 30 cases with Gleason score > 7, 4 cases with Gleason score ≤ 7. 4. Highly differentiated neuroendocrine cancer **(** **Figures 3A–F****)**: it was characterized by the nest-like structure and uniform nucleus, and the cytoplasm was peppered with salt-like chromatin or eosinophilic granules. And immunohistochemistry showed neuroendocrine differentiation. Only two cases were matched, and both AR and PSA staining were negative. 5. Poorly differentiated neuroendocrine carcinoma: 35 cases were matched, 15 cases were AR positive, and nine cases were PSA positive. And among them, two cases were large-cell neuroendocrine carcinoma **(** **Figures 3G–L****)**, 22 cases were small-cell carcinoma **(** **Figures 3M–R****)**. The cell morphological characteristics of small-cell carcinoma was similar to that of lung small-cell carcinoma, including delicate nuclear chromatin, little cytoplasm, lack of obvious nucleoli, and occasional necrosis, expression of neuroendocrine markers or not. In addition, there were some tumors whose histological morphology was intermediate cell type between small-cell carcinoma and prostate acinar adenocarcinoma, with relatively more cytoplasm and occasionally visible small nucleoli, and showed neuroendocrine differentiation, these cases were also included in this group. We did not include cases of Paneth cell-like differentiation.

### Clinicopathological Features of Usual CRPC and T-NEPC

T-NEPC showed a significant association with AR and PSA negative expression (*P<* 0.001) than Usual CRPC, there was no significant difference in Age, higher Gleason score, metastasis and TNM stage between two groups **(** **Table 1****)**. In T-NEPC patients, poorly differentiated neuroendocrine carcinoma are more prone to AR and PSA negative expression than prostate adenocarcinoma with neuroendocrine differentiation(*P<*0.001), there was no significant difference in clinicopathological features between prostate adenocarcinoma with diffuse neuroendocrine differentiation and prostate adenocarcinoma with focal neuroendocrine differentiation except AR positive(*P* = 0.034) **(** **Table 2****)**.

**Table 1:** Comparison of clinicopathologic findings of Usual CRPC Versus T-NEPC.

Characteristics	Usual CRPC (N = 137)	T-NEPC (N = 94)	P-value
Age (y)			
<65	42 (30.7%)	25 (26.6%)	0.556
≥65	95 (69.3%)	69 (73.4%)	
Gleason score			
≤7	33(24.1%)	11 (19.3%)	0.573
>7	104(75.9%)	46 (80.7%)	
Metastasis			
No	62 (45.3%)	37 (39.4%)	0.418
Yes	75 (54.7%)	57(60.6%)	
AR			
Negative	12 (8.8%)	29 (30.9%)	**0.000**
Positive	125 (91.2%)	65 (69.1%)	
PSA			
Negative	24 (17.5%)	45 (47.9%)	**0.000**
Positive	113 (82.5%)	49 (52.1%)	
TNM stage			
Stage I–II	47(34.3%)	29(30.9%)	0.312
Stage III–IV	90(65.7%)	65(69.1%)	

Gleason score of T-NEPC only evaluate the cases of prostate adenocarcinoma with neuroendocrine differentiation.

Bold values denote statistical significance at the p < 0.05 level.

**Table 2:** Comparison of clinicopathologic findings of T-NEPC.

Parameter	Pca-Focal NE (N = 23)	Pca-Diffuse NE (N = 34)	P-value	Pca-NE (N = 57)	NEC (N = 37)	P-value
Age (y)						
<65	6 (26.1%)	8 (23.5%)	0.559	16 (28.1%)	9 (24.3%)	0.812
≥65	17 (73.9%)	26 (76.5%)		41 (71.9%)	28 (75.7%)	
Metastasis						
Negative	10 (43.5%)	12 (35.3%)	0.587	22 (38.6%)	15 (40.5%)	1.000
Positive	13 (56.5%)	22 (64.7%)		35 (61.4%)	22 (59.5%)	
AR positivity						
Negative	0 (0%)	7 (20.6%)	**0.034**	7 (12.3%)	22 (59.5%)	**0.000**
Positive	23 (100%)	27 (79.4%)		50 (87.7%)	15 (40.5%)	
PSA positivity						
Negative	6 (26.1%)	11 (32.4%)	0.770	17 (29.8%)	28 (75.7%)	**0.000**
Positive	17 (73.9%)	23 (67.6%)		40 (70.2%)	9 (24.3%)	
TNM stage						
Stage I–II	9 (39.1%)	13 (38.2%)	1.000	22 (38.6%)	7 (18.9%)	0.067
Stage III–IV	14 (60.9%)	21(61.8%)		35 (61.4%)	30 (81.1%)	

Pca, prostate adenocarcinoma; NE, neuroendocrine differentiation; NEC, neuroendocrine cancer

Bold values denote statistical significance at the p < 0.05 level.

### The Clinical Outcome of Usual CRPC and T-NEPC

**Figure 4:**
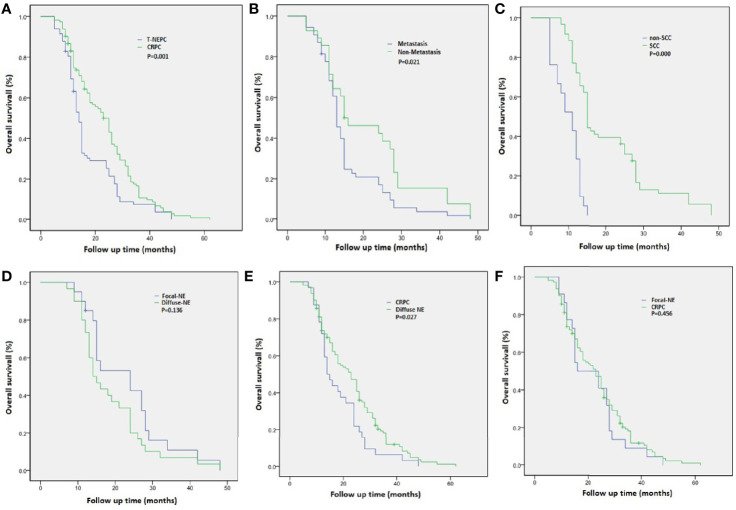
Overall survival (OS) from diagnosis of castration-resistant prostate cancer (CRPC) and treatment-emergent neuroendocrine prostate cancer (T-NEPC) in a cohort of patients with CRPC and T-NEPC. **(A)** OS in CRPC versus T-NEPC; **(B)** OS in T-NEPC patients with metastasis versus without metastasis; **(C)** OS in T-NEPC patients with small cell carcinoma component versus without small cell carcinoma component; **(D)** OS in patients with focal NE differentiation versus diffuse NE differentiation; **(E)** OS in patients with diffuse NE differentiation versus usual CRPC; **(F)** OS in patients with focal NE differentiation versus usual CRPC.

A total of 113 cases of usual CRPC and 82 cases of T-NEPC were followed up. There was a significant difference in OS between CRPC(OS from CRPC diagnosis, median OS 23.6 months,95% CI: 21.3–25.9 months) and T-NEPC(OS from T-NEPC diagnosis, median OS 17.6 months,95% CI: 15.3–19.9 months, log-rank *P* = 0.001) **(** **Figure 4A****)**. The median OS of T-NEPC patients without metastasis (median OS 21.4 months, 95% CI: 16.6–26.2 months) was significantly higher than that of patients with metastasis (median OS 15.7 months, 95% CI: 13.3–18.0 months, log-rank *P* = 0.021) **(** **Figure 4B****)**. In addition, we performed a comparison between T-NEPC subgroups. T-NEPC with small cell carcinoma component (median OS 9.7 months, 95% CI: 8.2–11.2 months) has a worse prognosis than without small cell carcinoma component (median OS 20.4 months, 95% CI: 17.6–23.3 months, log-rank *P* = 0.000) **(** **Figure 4C****)**. Prostate adenocarcinoma with diffuse NE differentiation (median, OS 18.8 months, 95% CI: 15.3–22.3 months) lacked prognostic significance compare with patients with focal NE differentiation (median OS 22.9 months, 95% CI: 18.1–27.7 months, log-rank *P* = 0.136) **(** **Figure 4D****)**. But prostate adenocarcinoma with diffuse neuroendocrine differentiation(median OS 18.8 months, 95% CI: 15.4–22.1 months) showed a statistically worse prognosis compare with usual CRPC (median OS 23.6 months,95% CI: 21.3–25.9 months, log-rank *P* = 0.027) **(** **Figure 4E****)**, while focal differentiation (median OS 22.9 months, 95% CI: 18.1–27.7 months) lacked prognostic significance compare with usual CRPC (median OS 23.6 months,95% CI: 21.3–25.9 months, log-rank *P* = 0.456) **(** **Figure 4F****)**. In unadjusted Cox regressions, an excess risk of overall death was observed in tumors with negative PSA (HR = 2.86; 95% CI = 1.39–6.73) and tumors with small-cell carcinoma (HR = 4.35; 95% CI = 1.76–7.36) **(** **Table 3****)**. However, AR negative, radiotherapy/chemotherapy, NE marker score and present of adenocarcinoma did not show an association with overall mortality.

**Table 3:** Univariate Cox regression analysis for overall survival in NEPC patients with a single molecular alteration as predictor of interest.

Characteristics	Hazard ratio (95% CI)	P value
AR Negative	0.85[0.59–1.73]	0.325
PSA Negative	2.86[1.39–6.73]	**0.019**
NE marker score	0.79[0.68–1.24]	0.098
Radiotherapy/Chemotherapy	1.64[0.81–3.16]	0.069
Present of small-cell carcinoma	4.35[1.76–7.36]	**0.001**
Present of adenocarcinoma	1.05[0.46–2.33]	0.798

Bold values denote statistical significance at the p < 0.05 level.

### Clinical Characteristics and Prognostic Factors of NEPC in the SEER Cohort

**Table 4** showed the characteristics of 476 NEPC cases, including 355 pure NEPC cases and 121 cases of prostate adenocarcinoma with NE differentiation. Compared total NEPC, pure NEPC were more likely to have PSA <2.5 ng/ml (29.86% vs 24.37%, **Table 4****)**. In multivariable-adjusted Cox models, an excess risk of overall death was observed in patients aged 65 years and older (HR = 1.35, 95% CI = 1.08–1.69), patients with PSA value ≤ 2.5 ng/ml (HR = 1.90, 95%CI=1.44–2.52), patients with PSA value 2.6–4.0 ng/ml (HR = 2.03, 95% CI = 1.38–2.99), tumor stage IV (HR = 2.13, 95% CI = 1.47–3.08) and other races than white and black (HR = 1.85, 95% CI = 1.17–2.94) in pure NEPC after adjustment for age at diagnosis, race, tumor stage, surgery treatment, and PSA levels, however no statistical significant was observed in those who diagnosed with prostate adenocarcinoma with NE differentiation **(** **Table 5****)**.

**Table 4:** Comparison of participant characteristics by histological subtype among 476 neuroendocrine prostate cancer from Surveillance, Epidemiology, and End Results (SEER) 18 registries, 2004–2017.

Characteristics	Total NEPC (n=476)	Pure NEPC (n=355)	NE differentiation (n=121)
	N (%) orMean (SE)^a^	N (%) orMean (SE)^a^	N (%) orMean (SE)^a^
**Age at diagnosis, mean, years**	68.28 (0.47)	68.31 (0.56)	68.20 (0.88)
**Age at diagnosis, years**			
<65	167 (35.08)	125 (35.21)	42 (34.71)
≥65	309 (64.92)	230 (64.79)	79 (65.29)
**Year of diagnosis**			
2004–2010	185 (38.87)	145 (40.85)	40 (33.06)
2011–2017	291 (61.13)	210 (59.15)	81 (66.94)
**Race**			
White	401 (84.24)	297 (83.66)	104 (85.95)
Black	46 (9.66)	35 (9.86)	11 (9.09)
Others	29 (6.09)	23 (6.48)	6 (4.96)
**Tumor TNM stage**			
Stage II	52 (10.92)	40 (11.27)	12 (9.92)
Stage III	16 (3.36)	10 (2.82)	6 (4.96)
Stage IV	390 (81.93)	289 (81.41)	101 (83.47)
Unknown	18 (3.78)	16 (4.51)	2 (1.65)
**Surgery, yes**	131 (27.52)	93 (26.20)	38 (31.40)
**PSA level**			
≤ 2.5 ng/ml	116 (24.37)	106 (29.86)	10 (8.26)
2.6–4.0 ng/ml	41 (8.61)	33 (9.30)	8 (6.61)
4.1–10.0 ng/ml	109 (22.90)	80 (22.54)	29 (23.97)
10.1–20.0 ng/ml	51 (10.71)	40 (11.27)	11 (9.09)
> 20.0 ng/ml	159 (33.40)	96 (27.04)	63 (52.07)
**Survival time, mean, months**	20.04 (1.22)	17.73 (1.34)	26.81 (2.68)

PSA, prostatic specific antigen; SE, Standard error; SEER, Surveillance, Epidemiology, and End Results.

^a^Presented as N and % (column percentage) or Mean and SE (standard error).

**Table 5:** Multivariable^a^ hazard ratios for overall mortality among 476 neuroendocrine prostate cancer from SEER 18 registries, 2004–2017.

Parameter	Total NEPC (n=476)				Pure NEPC (n=355)				NE differentiation (n=121)			
	Death number	HR	95% CI	P-value	Death number	HR	95% CI	P-value	Death number	HR	95% CI	P-value
**PSA level**												
≤ 2.5 ng/ml	97	**1.90**	**1.44**–**2.52**	**<.001**	89	**1.67**	**1.21**–**2.31**	**0.002**	8	1.54	0.69–3.42	0.29
2.6–4.0 ng/ml	35	**2.03**	**1.38**–**2.99**	**<.001**	28	**1.83**	**1.17**–**2.86**	**0.008**	7	2.30	0.97–5.45	0.06
4.1–10.0 ng/ml	80	1.09	0.81–1.47	0.57	63	1.10	0.77–1.57	0.60	17	0.828	0.45–1.53	0.55
10.1–20.0 ng/ml	34	1.10	0.74–1.62	0.65	31	1.32	0.86–2.04	0.21	3	–	–	–
> 20.0 ng/ml	114	Ref	–	–	72	Ref	–	–	42	Ref	–	–
**Age at diagnosis, years**												
<65	119	Ref	–	–	96	Ref	–	–	23	Ref	–	–
≥65	241	**1.35**	**1.08**–**1.69**	**0.008**	187	**1.35**	**1.05**–**1.74**	**0.02**	54	1.204	0.72–2.01	0.48
**Race**												
White	306	Ref	–	–	239	Ref	–	–	67	Ref	–	–
Black	33	1.17	0.81–1.69	0.40	26	1.04	0.68–1.58	0.02	7	1.447	0.63–3.32	0.38
Others	21	**1.85**	**1.17**–**2.94**	**0.009**	18	**2.17**	**1.30**–**3.62**	**0.003**	3	1.493	0.43–5.18	0.53
**Tumor TNM stage**												
Stage II	33	Ref	–	–	25	Ref	–	–	8	Ref	–	–
Stage III	5	–	–	–	4	–	–	–	1	–	–	–
Stage IV	309	**2.13**	**1.47**–**3.08**	**<.001**	241	**2.52**	**1.64**–**3.86**	**<.001**	68	1.174	0.54–2.58	0.69
**Surgery**												
Yes	103	1.17	0.92–1.48	0.20	78	1.24	0.94–1.63	0.13	25	1.196	0.71–2.02	0.50
No	256	Ref	–	–	204	Ref	–	–	52	Ref	–	–

a Multivariable models were adjusted for age at diagnosis, race, tumor stage, surgery treatment, and PSA levels. Bold values denote statistical significance at the p < 0.05 level.

## Discussion

The mechanism of T-NEPC was not clear. One explanation was that neuroendocrine tumor cells were resistant to hormonal therapies that target AR signaling. Therefore, ADT could inhibit the proliferation of ordinary prostate adenocarcinoma cells and relieved clinical symptoms of patients, while preserving and enriching neuroendocrine tumor cells. The use of potent AR signaling inhibitors could promote the transformation of adenocarcinoma in the CRPC state to T-NEPC in a clonal manner (7, 8). Some other research found that molecular events of T-NEPC such as TMPRSS2-ERG gene rearrangement and fusion genes were similar to prostate adenocarcinoma, suggested that T-NEPC originated from prostate adenocarcinoma and ADT enabled adenocarcinoma cells to develop into T-NEPC cells through the mechanism of adaptive response/tumor resistance (9, 10).

Currently, 2016 WHO classification of Tumours of the Urinary System and Male Genital Organs divided prostate cancers with neuroendocrine differentiation (11): usual adenocarcinoma with neuroendocrine differentiation, adenocarcinoma with Paneth cell-like neuroendocrine differentiation, carcinoid tumor, small-cell neuroendocrine carcinoma, and large-cell neuroendocrine carcinoma. Small-cell neuroendocrine carcinoma and large-cell neuroendocrine carcinoma are both poor-differentiated neuroendocrine carcinomas. What’s more, there was no significant difference in treatment between them, and there were some overlaps in morphology in some cases. In addition, prostate adenocarcinoma with diffuse neuroendocrine differentiation Gleason score 5 + 5 = 10 (grade group 5) showed overlapping features of large-cell carcinoma due to its morphological crossing (12). The prognosis of adenocarcinoma with Paneth cell-like neuroendocrine differentiation was completely different from that of T-NEPC, which may be just a morphological change and should not be classified as T-NEPC. Therefore, we recommended classification T-NEPC as adenocarcinoma with neuroendocrine differentiation, well-differentiated neuroendocrine carcinoma and poor-differentiated neuroendocrine carcinoma.

The 2019 WHO classification of digestive system tumors divided the neuroendocrine neoplasm (NEN) into two categories according to mitotic count and the Ki-67 index of hotspots: highly differentiated neuroendocrine tumor (NET) and poorly differentiated neuroendocrine caicinoma (NEC). NET includes NET G1, G2, and G3, which still retained high differential morphological features of NEN, and NEC included large-cell and small-cell carcinoma (13). However, compared with the tumors of the digestive system, the mitotic count and ki-67 index of prostate cancer were generally low, so it could not be classified according to the classification standard of gastrointestinal neuroendocrine tumors. Some study has found that Ki-67 was an independent prognostic predictor of prostate cancer. However, due to a low number of cases, the cut-off value to define a proliferation index of different grades of prostate neuroendocrine carcinoma was not established, and further studies were needed.

When prostate cancer patients were clinically resistant to hormonal therapy, presented with the clinical features such as carcinoid syndrome, lower urinary tract symptoms, osteolytic bone metastases, visceral metastases as progressive metastases on imaging (14). In addition, an increase of serum neuroendocrine markers CGA and NSE, or the low level or significantly elevated level of PSA was disproportionate to tumor progression, the possibility of T-NEPC should be considered (15). And it was recommended to re-biopsy the rapidly progressing lesion to exclude the diagnosis of T-NEPC. Urologists and pathologists should strengthen strategic communication, and pathologists should be reminded to perform immunohistochemical analysis of neuroendocrine markers. Due to the classic prostate adenocarcinoma can also showed different degrees of neuroendocrine differentiation, and scattered or clustered neoplastic neuroendocrine cells could appear in prostate cancer. So immunohistochemistry for neuroendocrine markers were not recommended for general use on prostate acinar adenocarcinoma. Therefore, the diagnostic rate of T-NEPC was also hovering at a low level. We suggested that for CRPC patients with repeated biopsy and metastatic Gleason score 5 + 5 = 10 after the failure of endocrine therapy, routine neuroendocrine markers (Syn, CgA, and CD56) examination should be performed to exclude the diagnosis of T-NEPC. Our results showed that focal neuroendocrine differentiation of prostate adenocarcinoma was not a prognostic factor. Due to the possibility of non-specific staining, in the absence of the typical histomorphological manifestations of T-NEPC, we suggested that T-NEPC pathological diagnosis should follow strict definition and combine with the use of multiple neuroendocrine markers and morphological features: at least 1 neuroendocrine marker with a diffuse (>50%) positive could be diagnosed. However, if the morphological features of typical small-cell carcinoma, we believed that it should be diagnosed as T-NEPC even if all the neuroendocrine indicators were negative. T-NEPC, especially small-cell carcinoma, tended to have AR and PSA negative, however, in adenocarcinoma with neuroendocrine differentiation, AR was often positive and PSA expression was weakened or negative. Recent studies on T-NEPC had shown that AR often positively expressed in tumor cells, but despite the AR protein expression in the nucleus, the transcriptional activity of AR was very low, which indicated that epigenetic mechanisms may inhibit AR function (16). Our study found that the negative PSA expression was an independent prognostic factor, these results were consistent with previous reports (17, 18). As T-NEPC and primary neuroendocrine prostate cancer showed similar molecular pathological characteristics (1), we validated our results using the SEER NEPC database. Our results showed that in pure NEPC, the prognosis of patients with low serum PSA level (≤4 ng/ml) were significantly worse than other PSA (>4 ng/ml) groups.

In summary, T-NEPC had highly invasive, rapid progress, and poor prognosis, and was related to resistance to ADT. Although the effect of chemotherapy was not very satisfactory, the progress of molecular diagnostic testing was expected to create favorable conditions for the development of precision medicine (19). Pathologists and clinicians should pay attention to the timely diagnosis and treatment of T-NEPC to improve the survival time of patients.

## Data Availability Statement

The raw data supporting the conclusions of this article will be made available by the authors, without undue reservation.

## Ethics Statement

This study was approved by the Ethics Committee of China Medical University. Written informed consent was obtained from all participants.

## Author Contributions

QZ contributed to the study design, data analysis, interpretation, and manuscript preparation. YH, YZ, DL, JM, and BH contributed to data interpretation and manuscript revision. XQ contributed study design and immunohistochemical evaluation, manuscript revision. All authors contributed to the article and approved the submitted version.

## Conflict of Interest

The authors declare that the research was conducted in the absence of any commercial or financial relationships that could be construed as a potential conflict of interest.
